# Malnutrition in the Outcome of Wound Healing at Public Hospitals in Bahir Dar City, Northwest Ethiopia: A Prospective Cohort Study

**DOI:** 10.1155/2021/8824951

**Published:** 2021-02-18

**Authors:** Netsanet Fentahun, Yeabsira Anteneh, Yonatan Menber

**Affiliations:** ^1^Department of Nutrition and Dietetics, School of Public Health, College of Medicine and Health Science, Bahir Dar University, Bahir Dar, Ethiopia; ^2^Felege Hiwot Comprehensive Specialized Hospital, Bahir Dar, Ethiopia

## Abstract

**Background:**

Poor nutritional status affects the normal process of the wound healing stage. There is limited evidence regarding the association between malnutrition and wound healing in Ethiopia.

**Objective:**

To assess the association between nutritional status and wound healing progress among adult individuals who had undergone abdominal surgery at Public Hospitals, Ethiopia.

**Methods:**

A prospective cohort study was conducted on 310 adult patients who had undergone abdominal surgery from August to December 2019. Data were collected using a standardized, structured, and pretested questionnaire. Anthropometric and serum albumin measurements were used to measure nutritional status. A multivariable Cox-regression analyses model was fitted to show the association between malnutrition and wound healing and *p* value < 0.05 was used to declare statistical significance value.

**Results:**

The cumulative incidence rate of good wound healing was 65.5% (95% CI: 60.0–71.0). Patients who had normal preoperative body mass index (adjusted hazard ratio (AHR) = 2.22 (95% CI: 1.55–3.19)) and normal range of serum albumin level (≥3.5) (AHR = 1.56 (95% CI: 1.05–2.29)) were significantly associated with better wound healing outcomes.

**Conclusion:**

Nutritional status had a strong association with good wound healing outcomes. Therefore, nutritional status screening should be done for all adult patients before undergoing abdominal surgery to improve wound healing outcomes and reduce hospital stays.

## 1. Background

The wound is defined as damage, disruption, or loss of the normal anatomical structure and functional continuity of living tissues accidentally, intentionally, or as a result of a disease process [1]. Wound healing is a continuing and complex process of changing injured tissue to another new tissue with the coordination of several biological and molecular events, which directly correlated to patients' overall nutritional status [2].

Poor nutritional status affects the normal process of the wound healing stage by prolonging the inflammatory phase, decreasing fibroblast proliferation, and altering collagen production which decreases wound tensile strength and increased infection rates [3, 4]. Previous studies found that wound is a common surgical problem all over the world that causes significant morbidity and mortality. Approximately 3–6 million people are affected by nonhealing wounds in the United States of America [5–7]. The incidence rate of the postoperative complicated outcome was 4–7% in Canada, 10.33% in Barcelona city, 38.8% in Sub-Saharan Africa, and 19.1% in Ethiopia from admitted surgical patients [8–11].

The patient with hypoalbuminemia who had undergone abdominoperineal resections was more likely to increase the risk of delayed wound healing as compared with the well-nourished [12]. While on serum albumin measurement operated surgical patients with normal nutritional status had a higher probability of wound healing than the counterpart [13]. The percentage of postoperative wound healing incidence rate was 11.40% from the healthy individuals but 2.30% in malnourished patients [14]. Based on serum albumin measure, the incidence rate of delayed wound healing was 12% and 28% from the well-nourished and undernourished, respectively. Based on body mass index (BMI) measure, the incidence rate of delayed wound healing was 12% and 21% from the well-nourished and undernourished, respectively [15].

Even though the postoperative complication rate of abdominal surgery patients in the Sub-Saharan African setting is still high [16], there is limited published evidence on the incidence of wound healing associated with the effect of nutritional status among patients undergoing abdominal surgery in Sub-Saharan African especially in Ethiopia. Also, there are no nutrition screening services in Ethiopia before undergoing abdominal surgery. This makes the outcome of abdominal surgery the worst and a long hospital in Ethiopia. Therefore, this study will be important to generate evidence on the association between malnutrition and wound healing progress among adult patients undergoing abdominal surgery in Ethiopia. Likewise, this study helps to change the usual trend of the integration of nutrition screening services with clinical management.

## 2. Methods

### 2.1. Study Design and Setting

Prospective cohort study design was conducted at Bahir Dar City Public Hospitals in Amhara Region, Ethiopia, from August to December 2019. Felege Hiwot Comprehensive Specialized Hospital (FHCSH), Tibebe Ghion Specialized Hospital (TGSH), and Addis Alem Primary Hospital (AAPH) were included in the study.

### 2.2. Study Population, Sample Size, and Sampling Procedure

The study population was adult patients (≥18 years) who underwent abdominal surgery at the surgical ward of Bahir Dar City Public hospitals. The sample size was calculated by considering 95% confidence level (CL), 80% of power (1-*β*), and taking nutritional status with1:1 ratio (well-nourished vs. malnourished) as exposure variable [14] and 10% nonresponse rate. The final sample size was 310 adult patients (155 for well-nourished and 155 malnourished patients) who had undergone abdominal surgery.

Three hundred ten adult abdominal surgical operation patients were selected by using a systematic sampling technique. The final sample size was proportionally allocated to the selected hospitals based on their last four-month abdominal surgical operation reports. The first case was selected by lottery method and then every three cases were included for both well-nourished and malnourished patients.

### 2.3. Data Collection Procedure and Quality Improvement

Data were collected using a standardized, structured, and face-to-face interviewer-administered questionnaire. The questionnaire includes the patient's sociodemographic characteristics, clinical features of patients, lifestyle and behavior of patients, and nutritional status which include the anthropometric examination and biochemical test. Data were collected by six trained B.Sc., Nurse and three trained medical laboratory technology with fluency in the local language (Amharic). Orientation, supervision, and reviewing the completed questionnaire were done to assure data quality. The data collectors measured the patient's preoperative nutritional status (BMI and serum albumin level) and followed postoperative wound healing progress with clinical features.

### 2.4. Anthropometric Measurements

Body weight was measured twice to the nearest 0.1 kg, and the average reading was calculated. While measuring the body weight, each patient was asked to take off the heavy clothes and shoes to keep in light clothes and remove heavy objects such as wallet and mobile before standing on the calibrated weighing scale balance (SECA, Germany) before the operation.

Height was measured twice by using SECA calibrated Stadiometer read as in centimeter. Each individual was asked to stand as fully erected as possible as against the wall and maintain the head in a position of straight-forward gaze and feet slightly apart with the back of the head, scapulae, buttocks, and heels positioned in contact with the wall; then, the sliding bar was lowered horizontally to the participants head to where the bar was locked and the measurement read and recorded during admission. Well-nourished patients' BMI were between 18.5 and 24.9 kg/m^2^, while malnourished patients' BMI were ˂18.5 kg/m^2^ (underweight) or ≥25 kg/m^2^ (overweight) [17].

### 2.5. Biochemical Test

Blood specimens were taken from sample patients before undergoing abdominal surgery. The medical laboratory technologists analyzed the serum albumin level as per the standard operating procedure. And then serum albumin level was classified as the normal range (>3.5 g/dl) and not the normal range (<3.5 g/dl) [17].

### 2.6. Wound Healing Follow-Up Checklist

All study patients were followed-up and their wound healing characteristics were assessed throughout the 1^st^, 3^rd^, 5^th^, 7^th^, and 10^th^ postoperative day. The data collectors estimated and recorded the wound healing progress by assessing sign and symptom of wound healing character (bleeding, scab formation, localized pain and swelling, persistent fever, red streaks near the cut, fouler odor, darkening skin edges, clear fluid, and thick grayish discharge around incision site) within ten postoperative days based on wound healing checklist. And also the level of the surgeon, regular wound care provider, patient's medication, privies surgical history, chronic comorbidity, mode, and type of surgery was assessed.

#### 2.6.1. Good Wound Healing

It includes patients who showed improvement regarding any sign of bleeding, swelling, localized pain, fever, redness, grayish or clear fluid discharge, and fouler odor, with the incision site filled by repaired tissue with the sign of shiny and smooth scar formation within ten postoperative days.

#### 2.6.2. Poor Wound Healing

It includes patients who had any sign of bleeding, persistent fever, red streaks near the cut, increasing localized pain and swelling, fouler odor, darkening skin edges, and thick-grayish or clear fluid discharge from the incision site until the 10^th^ postoperative days [18]. Finally, the patients who had good wound healing outcomes within ten postoperative days are considered as an event, and patients who had poor wound healing progress and were lost to follow-up (died, medical against, referral, defaulter, and self-discharge) until the 10^th^ postoperative day was considered as censored.

### 2.7. Statistical Analysis

Data were checked manually for completeness and consistencies and then coded and entered into EPI Data version 3.1 software and exported to SPSS (statistical package for social science) version 23 software package for further analysis. Descriptive statistics were used to describe variables and summarize the data in the form of frequency, mean, standard deviation, cross-tabulation, table, and graph.

Multivariable Cox-regressions analysis models were used to determine the association between malnutrition and wound healing outcomes. The model assumption was checked by using Log (-Log) S (t) plots. Crude and adjusted hazard ratios with their 95% confidence interval (CI) were estimated, and *P* value less than 0.05 was considered as the significance level for associations between dependent and predictor variables.

## 3. Results

### 3.1. Sociodemographic Characteristics of Participants

A total of 310 patients had undergone abdominal surgery at Bahir Dar City Public Hospitals in Amhara Region. The mean ages of the patients were 39.7 ± 16.66 years (±SD). The majority (92.3%) of patients were Orthodox Christianity followers. More than half (59.4%) of the patients were from urban residency. 191 (61.6%) patients were married, and higher proportions (33.5%) of patients had attended primary education. More than half (57.4%) of patients were recruited from FHCSH (Table 1).

### 3.2. Patients` Lifestyle

21 (6.8%) patients smoke cigarettes, and 96 (31.0%) patients were drinking different types of alcohol. 178 (57.4%) were walking from home to the workplace on foot. 138 (44.5%) had handwashing practice before eating their meals, while 281 (90.6%) patients sometimes had handwashing practice after toilet. 188 (60.6%) patients used pipe water for drinking purposes. 14 patients (4.5%) were doing regular physical exercises (Table 2).

### 3.3. Nutritional Status of Patients

According to BMI measurement, 155 (50.0%) preoperative patients were malnourished (48.4% underweight and 1.6% overweight). According to serum albumin level measurement, 171 (55.2%) preoperative patients were well-nourished.

### 3.4. Clinical Feature of Patients

159 (51.3%) patients were carried out with an emergency operation table. The most frequent type of abdominal surgery was laparotomy (39.4%). 242 (78.1%) were operated on by a senior physician. 148 (47.7%) patients have developed a disease process before two weeks, whereas 39 (12.6%) patients had a history of chronic comorbidity (Table 3).

### 3.5. Outcomes of Wound Healing

The overall incidence rate of good wound healing was 65.5% (95% CI: 60.0–71.0). From normal preoperative BMI patients, 88.4% had a good wound healing incidence rate whereas malnourished preoperative patients, 42.6%, had a good wound healing incidence rate. Likewise, from well-nourished preoperative patients in serum albumin measurement, 86.0% had good wound healing outcomes. Preoperative malnourished patients in serum albumin measurement had 40.3% of good wound healing outcomes.

### 3.6. Malnutrition and Wound Healing Outcomes

Before fitting the covariate into the model, the proportional hazard assumption was checked using Log (-Log) S (t) plots. The final model was adjusted with the type of medication, type of surgery, acute illness, chronic comorbidity, smoking, age of patients, and sex to know the association between malnutrition and wound healing outcomes. In the multivariable Cox regression analysis model, BMI and serum albumin test level were significant predictors of wound healing among adult patients who underwent abdominal surgery.

Patients who had the normal preoperative body mass index were 2 times more likely to have good wound healing incidence rate outcomes as compared to those malnourished patients (AHR = 2.22 (95% CI: 1.55–3.19)). Patients who had a normal range of serum albumin level (>3.5) were 1.6 times more likely to have good wound healing status as compared to malnourished patients (AHR = 1.56 (95% CI: 1.05–2.29)) (Table 4).

## 4. Discussion

Wound healing is a complex and dynamic process that needs well-nutritional status for the rapid inflammatory phase, promote collagen production, enhance fibroblast proliferation, and fast granulation to increase wound tensile strength for good wound healing outcome. Preoperative well-nutritional status is a fundamental aspect for optimal wound healing which decreases hospital length of the stay, minimizes health care cost, and reduces postoperative infection and complication rate [19]. As the finding of this study, out of 310 adult patients who had undergone an abdominal surgical procedure, 209 patients were improved from surgical incision which gives 65.0% with 95% CI: 0.60–0.71 of overall good wound healing incidence rate. The finding was similar to studies done in India 63% [15], Brazil 67.8% [13], and Gaza Strip 65% [20], but lower compared to studies done in Korea 73% [21] and Italy 90% [22], the discrepancy might be due to difference of socioeconomic status, the magnitude of sample size, distribution of geographical area, and different sit of the incisional wound and mode of surgery (only elective or emergency or both elective and emergency surgery).

In this study nutritional status was found to be significantly associated with wound healing outcomes. This result was in agreement with other studies done in India [14], Italy [22], India [15], and Korea [21]. This finding could be explained by the fact that the patients had a good nutritional status which enhances the growth hormone and collagen production that increase tensile strength and decrease risk factor of wound healing dalliance.

While observing the actual practice in Ethiopian health facilities, there are no nutrition screening services before undergoing abdominal surgery. This makes the outcome of abdominal surgery worse and a long time hospitalization hospital. This study shows nutrition screening before undergoing abdominal surgery is an important point to improve wound healing outcomes and shorting hospitalization.

This study does not consider the effect of postoperative hospital-acquired malnutrition during a hospital stay because of the difficulty to measure the postoperative nutritional status of patients. This current follow-up study did not follow up those patients once who had good wound healing outcomes and discharge even if the wound relapses and develops any sign of poor wound healing at home. In this study, the patient's dietary intake habit and immunity do not include a predicted variable due to difficulty to measure. Despite limitations, this study provides vital baseline information on incidence rate and predictors of wound healing among adult patients who had undergone abdominal surgery at Bahir Dar City Public Hospitals, Northwest Ethiopia.

## 5. Conclusion

Nutritional status had a strong association with good wound healing outcomes. Therefore, nutritional status screening should be done for all adult patients before undergoing abdominal surgery to improve wound healing outcomes and reduce hospital stays.

## Figures and Tables

**Table 1:** Sociodemographic characteristics of adult patients who had undergone abdominal surgery at Bahir Dar City Public Hospitals, Northwest Ethiopia, 2019 (*N* = 310).

Sociodemographic character	Categories	Frequency	Percentage
Study settings	FHCSH	178	57.4
	TGSH	88	28.4
	AAPH	44	14.2

Age in year	18–40	173	55.8
	41–65	93	30.0
	>65	44	14.2

Sex	Male	184	59.4
	Female	126	40.6

Religion	Orthodox	286	92.3
	Others^a^	24	7.7

Marital status	Married	191	61.6
	Single	82	26.5
	Divorced	20	6.5
	Widowed	17	5.5

Education	No formal education	50	16.1
	Primary	104	33.5
	Secondary	75	24.2
	More than secondary	81	26.1

Occupation	Farmer	98	31.6
	Merchant	75	24.2
	Employed	64	20.6
	Student	29	9.4
	Others^b^	25	8.1

Residency	Urban	184	59.4
	Rural	126	40.6

^a^Muslim and protestant,^b^daily labor, retired, housewife; FHCSH: Felege Hiwot Comprehensive Specialized Hospital; TGSH: Tibebe Ghion Specialized Hospital; AAPH: Addis Alem Primary Hospital.

**Table 2:** Lifestyle of adult patients undergoing abdominal surgery at Bahir Dar City Public Hospitals, Northwest Ethiopia, 2019 (*N* = 310).

Variables	Categories	Frequency	Percentage
Cigarette smoking	Yes	21	6.8
	No	289	93.2

Duration of smoking (*n* = 21)	Daily	8	2.6
	Occasionally	13	4.2

Alcohol consumption	Yes	96	31.0
	No	214	69.0

Frequency of alcohol consumption per day (*n* = 96)	≤three times a day	62	20.0
	>three times a day	34	11.0

Regular physical exercise	Yes	14	4.5
	No	296	95.5

Duration of physical exercise (*n* = 14)	≤three times a week	14	4.5
	>three times a week	0	0.0

Means of transport from home to workplace and workplace to home	On foot	178	57.4
	On transportion	132	42.6

Source of drinking water	Pipe water	188	60.6
	Springwater	98	31.6
	Well water	24	7.7

Handwashing after toilet	Always	29	9.4
	Sometimes	281	90.6

Handwashing before eating	Always	138	44.5
	Sometimes	172	55.5

**Table 3:** Clinical features of adult patients who had undergone abdominal surgery at Bahir Dar City Public Hospitals, Northwest Ethiopia, 2019 (*N* = 310).

Variables	Categories	Frequency	Percentage (%)
Type of current surgery	Laparotomy	122	39.4
	Herniorrhaphy	60	19.4
	Appendectomy	73	23.5
	Cholecystectomy	36	11.6
	Others	19	6.1

Level of an intervening surgeon	IESO	21	6.8
	Resident	47	15.2
	Senior	242	78.1

Mode of surgery	Elective	151	48.7
	Emergency	159	51.3

Duration of the disease process	<Two weeks	162	52.3
	≥Two weeks	148	47.7

Acute illness in the last 1 month	Yes	115	37.1
	No	195	62.9

History of chronic comorbidities	Yes	39	12.6
	No	271	87.4

Type of chronic comorbidities	Malignancy	17	5.5
	Others^h^	22	7.1

History of surgical intervention	Yes	8	2.6
	No	202	97.4

Type of patients current medication	NSAIDS	62	20.0
	Antibiotic	22	7.1
	Both	226	72.9

^g^Excision and achalasia, ^h^tuberculosis and diabetic mellitus, HIV/ADIS, IESO: Integrated Emergency Surgical Officers.

**Table 4:** Predictors of wound healing among adult patients undergoing abdominal surgery at Bahir Dar City Public Hospitals, Northwest Ethiopia, 2019 (*N* = 310).

Variables	Wound healing status			
	Good (%)	Censored (%)	CHR (95%CI)	AHR (95% CI)
*Body Mass index#*				
Malnourished	66 (42.6)	89 (57.4)	1	1
Well-nourished	137 (88.4)	18 (11.6)	3.18 (2.37–4.29)	2.22 (1.55–3.19)^*∗∗∗*^

*Serum albumin#*				
<3.5	56 (40.3)	83 (59.7)	1	1
≥3.5	147 (86.0)	24 (14.0)	3.16 (2.31–4.30)	1.56 (1.05–2.29)^*∗*^

#: adjusted with the type of medication, type of surgery, acute illness, chronic comorbidity, smoking, age of patients, and sex; ^*∗*^= *P* value <0.05 and ^*∗∗∗*^= *P* value ≤0.001; CHR: crude hazard ratio; AHR: adjusted hazard ratio.

## Data Availability

The datasets supporting the conclusions of this article are included in the article.

## Abbreviations

AAPH: Addis Alem Primary Hospital
AHR: Adjusted hazard ratio
BMI: Body mass index
CL: Confidence level
CHR: Crude hazard ratio
DM: Diabetes mellitus
FHCSH: Felege Hiwot Comprehensive Specialized Hospital
TGSH: Tibebe Ghion Specialized Hospital
WHO: World Health Organization.
